# Effects of Flaxseed and Its Components on Mammary Gland MiRNome: Identification of Potential Biomarkers to Prevent Breast Cancer Development

**DOI:** 10.3390/nu11112656

**Published:** 2019-11-04

**Authors:** Amel Taibi, Zhen Lin, Rong Tsao, Lilian U. Thompson, Elena M. Comelli

**Affiliations:** 1 Department of Nutritional Sciences, University of Toronto, Toronto, ON M5S 1A8, Canada; amel.taibi@utoronto.ca (A.T.); zn.lin@utoronto.ca (Z.L.); 2 Guelph Research and Development Centre, Agriculture and Agri-Food Canada, West Guelph, ON N1G 5C9, Canada; rong.cao@canada.ca; 3 Joannah and Brian Lawson Centre for Child Nutrition, University of Toronto, Toronto, ON M5S 1A8, Canada

**Keywords:** breast cancer, flaxseed, mammary gland, microRNAs

## Abstract

Breast cancer is the most common cancer among women worldwide. We previously showed that early-life exposure to flaxseed (FS) or its components, FS oil (FSO) and secoisolariciresinol diglucoside (SDG), affects the mammary gland (MG) and is associated with the reduction of breast cancer risk during adulthood. However, the underlying mechanisms are not understood. This study aimed to investigate the effect of FS, FSO, and SDG on the MG miRNA signature at a late stage of development. Female C57BL/6 mice, 4–5 weeks of age, were randomized into four groups to receive: (i) basal AIN-93G, (ii) 10% FS, (iii) 3.67% FSO, or (iv) 0.15% SDG. After 21 days, the mice were sacrificed and MG miRNAs were profiled. Diet-specific MG miRNA signatures were identified. Deregulated miRNAs were associated with breast cancer and targeted genes involved in MG development, growth, and cancer. The study allowed for the identification of potential biomarkers or novel therapeutic targets to prevent and/or reduce the risk of breast cancer.

## 1. Introduction

Breast cancer is the second leading cause of cancer death in female patients after lung cancer [1,2]. Thus, much research is being conducted for its prevention and treatment, including the role of diet. Because of its unique composition, flaxseed (FS) has been studied for its effect on breast cancer (reviewed in [3]).

Flaxseed (FS; *Linum usitatissimum*) is an oilseed containing approximately 40% oil (FSO), more than half of which is the n-3 polyunsaturated fatty acids (PUFA), α-linolenic acid (ALA), the highest level of the dietary phytoestrogen lignan, secoisolariciresinol diglucoside (SDG) (~1%), and approximately 30% dietary fiber [4]. Due to the elevated contents of ALA, FSO is considered to be an anti-inflammatory agent, while the lignan components have documented anti-oxidant and estrogenic/anti-estrogenic activities. Dietary fiber is generally recognized as having many beneficial effects, including modulation of the gut microbiota with the production of short-chain fatty acids (SCFA) [5].

We previously showed that FS consumption was associated with the reduction in breast cancer risk (reviewed in [3]). In mice injected with breast tumor cells, 10% FS significantly decreased the tumor size and growth at a late stage of carcinogenesis [6]. The 10% FS-induced inhibition of breast cancer growth and metastasis was related to the downregulation of insulin-like growth factor 1 and epidermal growth factor [7]. The FS inhibitory effect on breast cancer was also attributed to both FSO and SDG components.

In addition, studies from our group showed the link between early-life exposure to FS, mammary gland (MG) development, and breast cancer risk during adulthood. Notably, a 10% FS exposure in utero, during suckling, and throughout life could improve MG morphogenesis and differentiation, which in turn, helps to prevent MG carcinogenesis (reviewed in [3]). In addition, SDG-based diet during pregnancy and lactation helped in reducing the susceptibility of mice to carcinogenesis and reduced tumorigenesis. This pattern was not observed with FSO-based dietary intervention [8]. Taken together, these findings support the use of FS and SDG during MG development to reduce the future risk of breast cancer. However, the mechanisms involved in the protective effects of early-life exposure to FS and its components remain unclear. These may involve gene expression regulation via microRNAs (miRNAs).

MiRNAs are a class of small non-coding RNAs that regulate gene expression at the post-transcriptional level [9]. MiRNAs are involved in several physiological processes across multiple tissues, including playing a vital role during MG development through the regulation of cell differentiation, proliferation, and involution across different stages of life [10,11]. Their aberrant expression levels were associated with cancers, including breast cancer (reviewed in [12]). In a recent systematic review, potential markers of breast cancer were identified for early detection and diagnosis, including miR-210 and miR-21 [13].

Interestingly, environmental factors such as diet have been shown to alter the expression of miRNAs. Recent studies have showed that dietary phytochemicals may inhibit breast cancer through the regulation of miRNAs expression (reviewed in [14]). We recently found that n-3 PUFA altered the expression of miR-21 in MCF-7 breast cancer cells [15]. To date, no studies have investigated the effect of FS and its components, FSO and SDG, on the miRNA expression signature during MG development. Understanding the functions and contextual interactions between dietary components and MG miRNA signature (miRNome) during development could provide insights on the mechanism employed by bioactive agents to generate positive health outcomes and subsequently mitigate disease risk for lifelong health.

The objective of this study was to investigate the effect of exposing female C57BL/6 mice to 10% FS, as well as FSO or SDG found at the level in 10% FS, during MG development on MG miRNA expression signature and to identify potential targets to prevent breast cancer development during adulthood.

## 2. Materials and Methods

### 2.1. Animal Study and Dietary Treatments

Fifty-six C57BL/6 female mice were purchased from Charles River Laboratories (Senneville, QC, Canada) at 4–5 weeks of age in order to ensure the mammary glands (MGs) were entering the pubertal stage of development. Upon arrival, the mice were randomized into four groups (*n* = 14/group) and maintained on a basal modified AIN93G diet ([16], BD) for 7 days acclimatization. The AIN93G diet was modified to increase the fat percentage from 7% to 20%, as in our previous studies [17]. Due to its low n-3 PUFA and phytosterol content, corn oil was used instead of soybean oil to minimize the confounding effect that may otherwise derive from soybean oil. Mice were housed four per cage at 21 °C and on a 14:10 light/dark cycle with HEPA (high efficiency particulate air) filtered air. On day 0, all mice were inspected for vaginal opening to confirm the onset of puberty and then fed either (a) BD, (b) 10% FS, (c) 3.67% FS oil (FSO), or (d) 0.15% SDG diets for 3 weeks until sacrifice (Day 0 to 21). The FSO and SDG were at the same levels present in the FS diet.

Omegalo^®^ Cold Milled FS and FSO, kindly provided by Omega Nutrition Inc. (Vancouver, BC, Canada), were used to supplement the 10% FS and 3.67% FSO diets, respectively. SDG (99.5% pure) was prepared as previously described [18]. The FSO fatty acid composition was analyzed using gas chromatography by Omega Nutrition. The proximate composition of FS was analyzed by Maxxam Analytics (Mississauga, ON, Canada), while SDG content was analyzed using HPLC by ChromaDex Analytics (Boulder, CO, USA) Table S1). Diets were formulated based on the AIN-93G diet (Table S2) using the FS compositional analysis and were prepared by Dyets Inc. (Bethlehem, PA, USA). The diets were isocaloric, with equal macronutrient levels. Diets were stored at 4 °C until needed. Diets in cages were replaced every 2 to 3 days.

Food intake and body weight were measured twice a week. At day 21, mice were sacrificed by cervical dislocation and blood was collected via cardiac puncture in a serum vacutainer for lignan analysis. The fourth left MG was dissected and immediately stored at −80 °C for further analyses.

All the animal procedures were performed in accordance with the Regulations of the Animals for Research Act in Ontario and the Guidelines of the Canadian Council on Animal Care, and were approved by the animal ethics committee of the University of Toronto (Animal Use Protocol #: 20011734).

### 2.2. Serum Lignan Analysis

Serum concentrations of the lignans enterodiol (END), enterolactone (ENL), and secoisolariciresinol (SECO) were determined by liquid chromatography-mass spectrometry (LCMS/MS) as previously described [19].

### 2.3. NanoString nCounter miRNA Profiling and Data Analysis

Total RNA was extracted from the MG samples (*n* = 6/group) using the mirVana^TM^ miRNA isolation Kit (Ambion, Life Technologies, Waltham, MA, USA). RNA concentration and purity were measured using the NanoDrop ND-2000 (ThermoScientific, Wilmington, DE, USA).

The expression profile of 578 miRNAs was assessed with the nCounter^®^ Mouse v1.5 miRNA Expression Assay Kit (NanoString Technologies, Seattle, WA, USA) (miRBase built v15). The experiments were performed at the Princess Margaret Genomics Centre Toronto, Canada (www.pmgenomics.ca), using 200 ng of total RNA according to the manufacturer’s instructions.

Data analysis was performed using nSolver^TM^ Analysis Software v4.0 (NanoString Technologies). Background hybridization was corrected by subtracting the geometric mean calculated from eight negative controls. Data were then normalized to the geometric mean of all probes. To filter for expressed miRNAs, only probes that were above the background in 80% of the samples of any of the treatment groups were retained. Samples with less than 75% probes above the background were excluded. The final dataset was log2 transformed and used for statistical analysis.

Unsupervised hierarchical clustering analysis was performed with the normalized and log2 transformed data using ClustVis (https://biit.cs.ut.ee/clust vis/) [20].

### 2.4. MiRNAs Target Prediction and Pathway Analysis

Experimentally validated target genes of differentially expressed miRNAs were identified in silico using the miRWalk Release 2.0 and miRTarBase Release 7.0 databases in order to include all validated target genes that overlapped between and were exclusive to the two databases [21,22].

Gene enrichment analysis was performed for miRNA gene targets using one-sided hypergeometric test conducted on Gene Ontology (GO) biological process (GObp), molecular function (GOmf), and cellular component (GOcc) gene sets using ClueGO, a Cytoscape plug-in application [23]. The GO gene sets with a Benjamini–Hochberg adjusted *q* < 0.05 were considered significant and were clustered into ClueGO groups based on the similarity in the number of genes calculated by a Kappa score within ClueGO. Data were visualized using Cytoscape (v3.7.1) [24].

### 2.5. Statistical Analysis

Statistical analyses were performed using GraphPad Prism 6.0 (GraphPad Software, Inc., La Jolla, CA, USA). Data are presented as means ± standard error of mean (SEM). To analyze body weight and food intake, one-way ANOVA with Bonferroni’s post-hoc for multiple comparisons test was used.

Serum lignan concentration was analyzed by Kruskal–Wallis test with Dunn’s post-hoc for multiple comparisons and differences were considered significant with *p* ≤ 0.05. For miRNA, one-way ANOVA was used to calculate statistical significance of the normalized log2-transformed miRNA expression counts between the treatment groups. The *p*-values were corrected using the Benjamini–Hochberg method (FDR < 0.2). Pairwise comparisons were used to compare between the treatment groups.

## 3. Results

### 3.1. Body Weights and Food Intake

Body weights and food intake did not differ among the treatment groups (Table S3).

### 3.2. Serum Lignan Concentration

Serum concentrations of END, ENL, and SECO were detectable in the FS and SDG groups but not in the BD and FSO groups (Table S4). Multiple comparison analysis showed significant differences in SECO, END, ENL, and total lignan concentrations between the 10% FS and the BD- or FSO-fed groups. Significant differences in SECO and ENL concentrations were found between the SDG-fed group and the BD- or FSO-fed group. No significant differences were found between the 10% FS group compared to the SDG group despite relatively much higher lignan levels in the 10%FS group due to large variability, particularly in the FS group.

### 3.3. Mammary Gland miRNome

**Figure 1:**
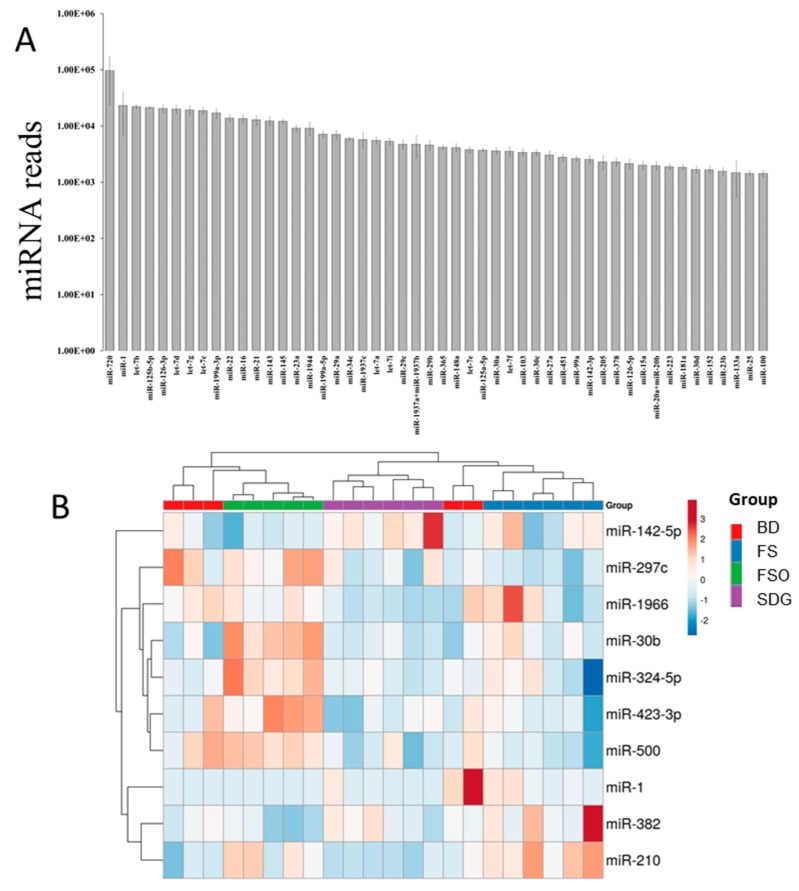
Mammary gland (MG) miRNAs changes in response to the three experimental diets. (**A**) Top miRNAs detected in MG using NanoString Technologies (mean values ± SEM); (**B**). Heatmap showing unsupervised hierarchical clustering of the 10 significantly deregulated miRNAs between the treatment groups (*p* value < 0.05, FDR < 0.2), *n* = 5–6/group. For each miRNA, the expression values were transformed to *Z*-scores, where red indicates higher expression and blue indicates lower expression.

Among the 578 miRNAs profiled in MG, 238 were detected (Table S5), with 45 of the top 50 expressed miRNAs shared between the groups (Figure 1A). After applying the cut-off, one sample from the BD group and another from the FSO group were excluded. Ten miRNAs were found to be significantly differentially expressed between the treatment groups (*p* < 0.05, FDR < 0.2). Unsupervised hierarchical clustering based on the expression profiles of these miRNAs produced a separation between samples from different treatment groups, even if not perfect (Figure 1B). This separation was clear when using the three groups (FS, FSO, and SDG) to construct the heatmap (Figure S1).

Pairwise comparisons showed a diet-specific effect on miRNAs in the MG (Figure S2). In the FS group, both miR-297c and miR-500 were downregulated compared to the BD and FSO groups, while miR-1 was upregulated compared to the FSO, with no significant change compared to the BD. In addition, the 10% FS diet significantly increased the expression of miR-210 compared to the BD and the SDG diets.

FSO significantly deregulated the expression of four miRNAs: miR-30b, miR-324-5p, miR-382, and miR-423-3p. MiR-30b and miR-324-5p were significantly increased by FSO compared to BD and SDG. While miR-382 and miR-423 were significantly down- and upregulated, respectively, compared to the FS group, their expression levels were not significantly changed compared to the BD group (Figure S2).

The SDG diet, in turn, significantly increased miR-142-5p and decreased miR-1966 expression levels compared to the BD and FSO groups.

### 3.4. Identification of Gene Targets of Deregulated miRNAs and Pathway Enrichment Analysis

In silico analysis using miRWalk Release 2.0 and miRTarBase Release 7.0 databases identified a total of 162 experimentally validated genes (Table S6).

To identify the pathways enriched in response to each treatment, we completed the analysis using the list of validated targets of miRNA significantly changed in FS, FSO, and SDG groups separately. Using the Cytoscape plug-in application ClueGO, we identified significantly enriched GO gene sets, which were grouped together based on common function.

**Figure 2:**
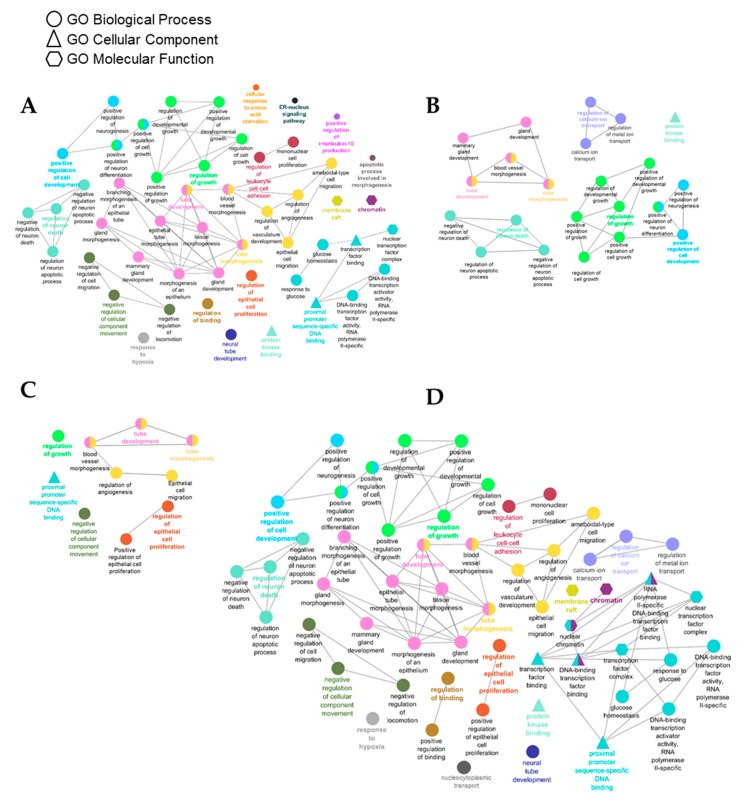
Functional enrichment analysis of miRNA target genes. Network analysis for target genes of miRNAs deregulated in FS (**A**), FSO (**B**), SDG (**C**), and for all validated targets identified in the study (**D**). The enrichment analysis was performed across gene ontology (GO) molecular function (GOmf), biological process (GObp), and cellular component (GOcc) pathways. Significantly enriched genes (Benjamini–Hochberg (HB) adjusted *q* < 0.01) were used to construct the network. Network node layout is based on the similarity between the genes within the GO gene sets. Terms with a similarity score >0.5 were linked by an edge. Nodes of the same color belong to the same cluster.

The FS group had the highest number of sets significantly enriched for the genes targeted by miR297c, miR-500, miR-1, and miR-210 (Figure 2A). These 47 GO gene sets were clustered into 17 CluGo groups, with the top five most significant GO terms being as follows: regulation of growth, regulation of cell development, tube development, tube morphogenesis (GObp), and protein kinase binding (GOmf).

In both, FSO and SDG groups, 22 and 10 gene sets were identified, respectively; all of them were identified in the FS and are shared between the groups, including regulation of growth, tube morphogenesis, and tube development (Figure 2B,C).

**Figure 3:**
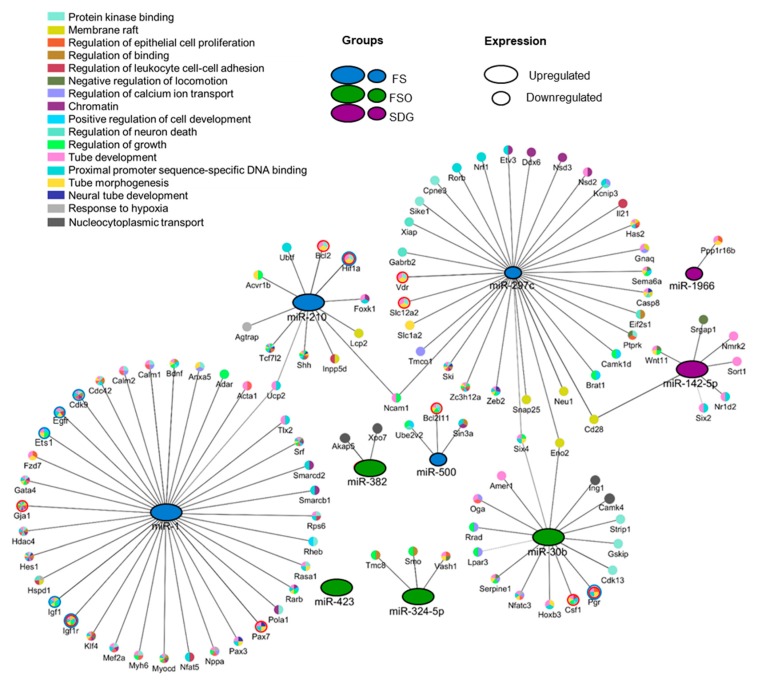
Regulatory network of miRNAs and their experimentally validated targets in enriched pathways (miR-423 is excluded due to no significantly enriched genes having been identified). Gene Ontology GOmf, GObp, and GOcc were used to color the nodes. The network was generated using Cytoscape 3.7.1 and consisted of the miRNAs deregulated in FS (green), FSO (blue), and SDG (purple), with the miRNA node size representing the expression level in the specific group. Nodes with red circles correspond to genes involved in MG development. Nodes with blue circles correspond to genes associated with breast cancer.

The miRNA-mRNA network shows that significantly deregulated miRNAs in the FS and FSO groups target genes involved in the MG development (red circles) (Figure 3) and/or associated with breast cancer (blue circles), including *Egfr, Igf-1, Igf-1R, Hif1a*, *Ets1*, and *pgr*.

## 4. Discussion

To investigate mechanisms underlying beneficial effects of FS in the context of breast health, we analyzed miRNA responses in the MG of pubertal mice to two main FS components, FSO and SDG, versus FS. We found overlap in miRNA expression across the three diets, but three diet-specific miRNA signatures could be identified. Specifically, a four-miRNA signature (higher miR-1 and miR-210 and lower miR-297c and miR-500 expression) significantly associated with the FS diet, a different four-miRNA signature (higher miR-30b, miR-324-5p and miR-423-3p and lower miR-382 expression) significantly associated with the FSO diet, and a two-miRNA signature (higher miR-142-5p and miR-1966) significantly associated with the SDG diet. This shows that the effects of FS components on MG miRNA expression are not additive. Upon ingestion, FS and its components are processed in the intestine mainly through the action of the gut microbiota. The microbiota ferments FS fiber, producing short-chain fatty acids and gases, and converts SDG to the enterolignans ED and EL, which may travel to distal organs. Here, despite being fed the same amount of SDG, the 10% FS group had relatively higher serum enterolignans concentrations compared to SDG-fed group, in line with our previously published studies, showing significantly higher urinary enterolignan levels in female mice receiving 10% FS compared to SDG-fed mice [25,26]. The fiber in FS may have enhanced the conversion of SDG to enterolignans due to enhanced microbial fermentation.

In this study, we examined the MG miRNome at the end of the MG developmental period. MiRNAs are known as one of the regulators of MG development. MG miRNA profiling in C57BL/6 female mice during development showed a distinctive miRNA signature based on developmental stages [27]. This study identified seven temporally co-expressed clusters, which were enriched in miRNAs associated with breast cancer including, miR-210, miR-30b, and miR-21. The breast cancer-associated miRNAs may play a role in cell proliferation and invasion during normal MG development, but their expression may become deregulated in breast cancer.

Taken together, this shows that miRNA expression regulation is critical throughout various stages of life in the MG development. Alterations in MG during the early stages of development have been associated with increased susceptibility to breast cancer [28]. Exposures to environmental factors, including hormones, chemicals, and diets, could play an important role in increasing this susceptibility [28,29].

This is the first comprehensive study of the effects of FS as a whole food compared to its isolated components, FSO and SDG, on MG miRNA signature in healthy female mice during MG development. Our key findings include the identification of (1) diet-specific MG miRNA signatures, (2) diet-dependent deregulation of selected breast cancer-associated miRNAs, and (3) enriched gene sets targeted by these miRNAs in each dietary intervention group.

Interestingly, most of the miRNAs identified in this study were shown to be deregulated in several types of cancers, including in breast cancer.

In the FS group, miR-1 was upregulated. This miRNA is known as a tumor suppressor in breast cancer, but was significantly downregulated in breast cancer compared to non-tumor tissues [30]. Breast cancer patients with low miR-1 expression levels were found to have a poor survival time [31]. Moreover, previous studies suggest that upregulation of miR-1 beneficially counters several types of cancers by repressing the expression of *Egfr* (coding for an epidermal growth factor receptor) to inhibit cell proliferation, migration, and invasion [32,33,34,35]. Diet-induced restoration of miR-1 may have a potential therapeutic significance.

Increased miR-500 serum levels have been linked to hepatocellular carcinoma [36], while its downregulation was found to suppress lung cancer proliferation [37]. However, it is not known if this miRNA is deregulated in breast cancer. FSO-associated miR-500 downregulation could potentially protect against mammary gland carcinogenesis and breast cancer development.

MiR-297c inhibition in human or animal subjects was found to be correlated with the efficacy of NSAIDs (non-steroidal anti-inflammatory drugs) in cancer chemoprevention treatment. This miRNA has, however, been suggested for use as a marker to assess the effectiveness of treatment [38]. Interestingly, in our study, miR-297c expression was significantly decreased in FS-fed mice.

In the FSO group, with mice receiving the equivalent amount of 3.67% FSO as in the FS group, both miR-30b and miR-324-5p were upregulated compared to the BD and SDG groups, with no significant changes in the expression levels when compared to the FS group.

MiR-30b is a member of the miR-30 family, identified as tumor suppressors, which have frequently been found to be downregulated in different types of tumors, including breast cancers (reviewed in [30]). This miRNA was shown to be able to alter drug resistance in breast cancer. A previous study found that a 6-day treatment of breast cancer cell lines with trastuzumab (Herceptin) resulted in a significant upregulation of miR-30b. Trastuzumab-induced miR-30b expression could, in consequence, induce cell growth inhibition by targeting cyclin E2 (CCNE2) [39]. MiR-30b was also found to be involved in MG development; its upregulation in the tissue led to an impaired MG function and structure during lactation and involution [40].

In a recent study, miRNA profiling of triple-negative breast cancer (TNBC) tissues showed high miR-324-5p expression levels. This expression was significantly correlated with decreased overall survival in breast cancer patients [41].

MiR-382 was reported to be upregulated in the serum and plasma of patients with breast cancer and suggested to be used as a non-invasive marker for the diagnosis of breast cancer [42]. This miRNA was also shown to promote cell viability, invasion, and survival in breast cancer patients [43].

MiR-142-5p is one of the oncogenic miRNAs targeting cancer-related pathways. Its expression levels are significantly higher in breast cancer cell lines compared to controls [44]. This miRNA was also identified as a negative regulator of TGF-β signaling pathway and suggested to be used as a therapeutic target in breast cancer [44].

For the SDG-induced miR-1966, very limited data are available to describe the role of this miRNA in MG development and/or in cancers. A very recent study showed that miR-1966 is significantly downregulated in toosendanin-induced liver injury in mice [45].

Interestingly, six of the ten miRNAs identified in this study (with the exception of miR-210 in the FS group, miR-423-3p in the FSO group, as well as the SDG-induced miR142-5p and miR-1966), were expressed in the opposite direction compared to their expression in the breast cancer tissues and/or cell lines. Therefore, it is speculated that FS and FSO dietary interventions could be used either to antagonize breast cancer-associated alterations of selected miRNAs or to prevent their deregulation in the MG during development. Note that mice used in this study were 4–5 weeks old, which corresponds to late stages of pubertal development. This supports the notion of early-life dietary intervention, using FS, to mitigate the risks of mammary gland malignancies and breast cancer.

This study brings a new concept of diet-dependent MG response that is mediated by miRNAs. Our data discern the differences between the consumption of purified FS components, SDG and FSO, and whole FS on the MG miRNA signature. However, the mechanisms behind these specific responses are not clear. This is likely due to the gut-microbiota derived metabolites resulting from the ingestion of FS as whole food and its isolated FSO (3.67%) or SDG (0.148%) components.

Overall, the gut microbiota outcomes may have a significant consequence on the amount and types of metabolites entering the systemic circulation, which could in consequence influence the miRNA-mediated mammary gland response (MG). Though, given the complexity and because the effect of FS and its components, FSO and SDG, on the gut-MG axis has not been completely elucidated yet, gut microbial profiling in response to different dietary interventions is needed for a better understanding of the mechanisms driving our diet-associated significant miRNA signatures.

To further explore the role of miRNAs in mediating MG response to different diets, we identified a regulatory network of the deregulated miRNAs with their experimentally validated targets. Although distinctive miRNA signatures were associated with different diets, the in silico analysis revealed the presence of shared gene sets between the three groups. Growth regulation and tube morphogenesis were identified among the most significantly enriched pathways. Thus, it could be postulated that deregulated miRNAs may be involved in promoting growth properties such as cell proliferation and differentiation in the MG.

It is not surprising to see that the FS-fed group had the greatest number of enriched gene sets followed by the FSO and SDG groups. Compared to isolated FSO and SDG, the FS contains soluble and insoluble dietary fiber, digestible proteins, and lignans, which may also have either a direct or an indirect effect on the MG response.

Interestingly, enrichment analysis shows that FS- and FSO- dependent miRNAs are associated with gene sets involved in MG development, including *Bcl2l11, Csf1, Gja1, Hif1a, Igf1, Igf1r, Pax3, Pgr, Slc12a2,* and *Vdr.*

Finally, constructing the miRNA-mRNA interaction network served not only to visualize the interaction between miRNAs and their targets, but also to identify potential biomarkers or novel therapeutic targets to prevent and/or reduce the risk of breast cancers. Of these, FS-induced miR-1 may be a potential candidate. In addition to its association with MG development, this miRNA also targets genes involved in breast cancer development, including *Igf-1* and *Igf-1R* coding for insulin-like growth factor 1 and its receptor (reviewed in [46] and [47]), as well as *Cdk9* coding for cyclin-dependent kinase 9 (CDK9), involved in cell survival and the regulation of apoptosis. Cdk9 inhibitors are considered to be anticancer therapeutics [48].

## 5. Conclusions

This research is significant, as it provides evidence that FS and its FSO and SDG components have different physiological consequences, which should be considered when used as a preventive or therapeutic agent. With the current rise of dietary supplements, this study reinforces the importance of discerning the difference in health outcomes between whole foods compared to isolated components. Furthermore, by taking a global approach, it suggests a potential mechanism via miRNA for FS to impact developing MG, a mechanism that has only been marginally considered so far.

## Supplementary Materials

The following are available online at https://www.mdpi.com/2072-6643/11/11/2656/s1, Figure S1: Heatmap showing unsupervised hierarchical clustering of the 10 significantly deregulated miRNAs between FS, FSO and SDG groups, Figure S2: Log2 normalized counts of the 10 deregulated miRNAs in response to diets. Table S1: Omegalo® Cold Milled Flaxseed composition, Table S2: Experimental diets composition, Table S3: Effect of various treatments on body weight and food intake over 21 days, Table S4: Serum lignans concentration mice from the 4 experimental diet groups, Table S5: miRNAs detected in the MG, Table S6: Validated targets of the 10 deregulated miRNAs in the MG.
