# Supplementary material for: Effects of Flaxseed and Its Components on Mammary Gland MiRNome: Identification of Potential Biomarkers to Prevent Breast Cancer Development
Source: Nutrients. 2019 Nov 4;11(11):2656. doi: 10.3390/nu11112656 (PMC6893416; doi:10.3390/nu11112656)
Supplement: Supplementary file 1 [file nutrients-11-02656-s001.pdf]

nutrients

MDPI

Table S1. Omegalo® Cold Milled Flaxseed composition

Component	Percent
Fat	36.7
**Fatty acids**	
**16:0**	6.6
**18:0**	6.2
**18:1n-9**	21.9
**18:2n-6**	13.0
**18:3n-3**	50.8
Protein	17.6
Ash	3.1
Carbohydrates	36.6
Dietary Fiber	28.6
Moisture	6.0
Secoisolariciresinol diglucoside (SDG)	1.5

Proximate composition analysis was performed by Maxxam Analytics (Mississauaga, ON, Canada), Secoisolariciresinol diglucoside (SDG) quantification was performed by ChromaDex Analytics (Boulder, CO, USA), and fatty acid analysis of flaxseed was performed by Omega Nutrition Inc. (Vancouver, BC, Canada). Percentages are presented as wet (as-is) basis.

Table S2. Experimental diets composition

**Component**	**Basal**	**Flaxseed**	**Flaxseed Oil**	**Secoisolaricires inol diglucoside (SDG)**
Macronutrient (%w/w, % kcal)				
Protein	20.3, 17.0	20.3, 17.0	20.3, 17.0	20.3, 17.0
Carbohydrate	54.2, 45.3	54.2, 45.3	54.2, 45.3	54.2, 45.3
Fat	20.0, 37.6	20.0, 37.6	20.0, 37.6	20.0, 37.6
Component (g/kg)				
Casein	200.0	182.4	200.0	200.0
L-Cystine	3.5	3.5	3.5	3.5
Sucrose	100.0	100.0	100.0	100.0
Cornstarch	305.5	288.4	305.5	304.0
Dyetrose	86.3	86.3	86.3	86.3
Cellulose	50.0	21.4	50.0	50.0
Mineral Mix #210025	40.3	40.3	40.3	40.3
Vitamin Mix #310025	11.5	11.5	11.5	11.5
Choline Bitartrate	2.9	2.9	2.9	2.9
Corn Oil	200.0	163.3	163.3	200.0
Flaxseed	0.0	100.0	0.0	0.0
Flaxseed oil	0.0	0.0	36.7	0.0
SDG	0.0	0.0	0.0	1.5

Table S3. Effect of various treatments on body weight and food intake over 21 days.

Treatment	Initial weight (Baseline, day 0) g	Final weight (Sacrifice, day 21) g	Total food intake (g/mouse/day) g
**BD**	17.71 ± 0.30	20.93 ± 0.49	2.17 ± 0.06
**10% FS**	18.00 ± 0.21	20.96 ± 0.29	2.37 ± 0.04
**FSO**	17.64 ± 0.34	20.90 ± 0.44	2.23 ± 0.04
**SDG**	17.36 ± 0.23	20.96 ± 0.51	2.45 ± 0.08

BD = basal diet; FS = flaxseed; FS = flaxseed oil; SDG = secoisolariciresinol diglucoside.
Data are means ± SEM, n=14/group.

Table S4. Serum lignans concentration mice from the 4 experimental diet groups

Lignan	BD	10% FS	FSO	SDG	p-value
SECO (nM)	NDa	74 ± 67^{b}	NDa	39 ± 32^{b}	<0.0001
END (nM)	NDa	214 ± 121^{b}	NDa	7 ± 5ab	<0.0001
ENL (nM)	ND^{a}	116 ± 93^{b}	NDa	2 ± 0.0^{b}	<0.0001
Total (nM)	NDa	404 ± 281^{b}	NDa	47 ± 37^{ab}	<0.0001

END= enterodiol; ENL= enterolactone; SECO= secoisolariciresinol. Data are means ± SEM of n=6/group per metabolite measured. $^{a\text{-}b}$ Values with different letters within the same row are significantly different ($p < 0.05$) by Kruskal-Wallis test followed by Dunn’s multiple comparisons test. ND, non-detectable.

Table S5: miRNAs detected in the MG*

	BD		FS		FSO		SDG	
miRNAs	Mean	SD	Mean	SD	Mean	SD	Mean	SD
let-7a	5581.0	2139.4	5840.9	2545.0	7377.9	1051.9	5010.5	1416.4
let-7b	22232.1	5255.0	30908.3	14428.9	21562.3	5081.9	17528.1	2551.5
let-7c	18832.6	6260.0	21633.9	8741.2	23049.2	5638.0	14750.1	3885.3
let-7d	20156.0	8694.8	23679.3	5257.0	21186.7	6127.5	16458.1	1281.7
let-7e	3814.4	1232.2	4035.9	1232.5	3963.0	476.8	3138.7	613.7
let-7f	3562.6	1716.1	3635.1	2383.4	5536.0	1090.8	3342.0	860.4
let-7g	19400.4	8782.5	26025.0	3614.3	23156.3	3970.3	17738.3	2919.7
let-7i	5389.5	1971.8	7141.7	2514.0	5653.2	431.4	5474.7	463.8
miR-1	23264.5	40925.8	12632.3	12095.1	11506.1	26664.5	4608.0	9718.7
miR-100	1438.9	472.8	1588.8	571.9	1399.7	298.5	1303.6	421.5
miR-101a	147.1	90.5	145.9	70.6	236.6	65.9	140.8	58.1
miR-101b	167.7	73.7	186.1	75.1	250.5	54.4	172.9	33.5
miR-103	3412.7	1289.8	3457.6	1752.1	3878.9	755.8	2925.2	929.0
miR-106a+miR-17	1101.1	417.8	1156.7	566.6	1506.3	265.0	1166.4	140.6
miR-106b	868.4	395.5	914.8	278.4	1401.4	280.9	955.8	168.3
miR-107	181.9	59.4	168.7	107.9	246.9	53.6	155.5	58.8
miR-10a	372.4	78.3	417.3	74.6	360.1	58.3	358.5	111.2
miR-10b	637.3	160.4	697.4	234.6	561.5	89.2	582.4	173.0
miR-1186	314.4	79.7	272.7	71.6	270.1	21.6	257.6	24.7
miR-1191	23.7	28.3	7.4	7.0	37.2	39.4	17.0	20.6
miR-1196	19.0	11.8	53.3	81.5	38.8	38.9	51.8	51.2
miR-1198	46.3	13.7	65.3	48.0	61.7	20.2	50.4	14.0
miR-122	21.2	14.1	63.0	34.9	25.3	26.6	19.8	15.7
miR-1224	152.2	50.8	374.4	579.7	129.0	32.7	149.1	50.2
miR-125a-3p	178.9	88.7	126.7	47.7	234.0	73.4	134.2	47.7
miR-125a-5p	3737.4	631.6	3581.5	1617.9	3308.5	701.9	2903.6	917.3
miR-125b-3p	107.8	25.6	96.6	44.5	92.0	23.6	96.8	29.9
miR-125b-5p	21386.0	3380.7	19043.5	7969.2	17468.9	4342.8	17084.8	4430.7
miR-126-3p	20549.4	8216.3	25592.5	3583.8	19883.6	3052.4	19360.6	4966.1
miR-126-5p	2146.2	1042.7	2456.5	987.6	2148.0	290.9	1766.0	410.3
miR-127	332.9	63.9	393.8	179.5	258.8	106.9	231.7	86.4
miR-128	25.7	13.9	33.3	9.7	32.9	5.9	26.5	5.0
miR-129-3p	92.3	54.5	74.3	28.0	103.8	34.2	73.0	23.0
miR-130a	1406.2	455.8	1640.5	282.3	1318.1	298.2	1317.3	445.6
miR-130b	49.8	9.0	50.7	7.9	61.6	17.9	53.9	12.3
miR-132	197.0	68.3	218.7	61.5	181.2	59.5	156.4	17.3
miR-133a	1482.6	2331.3	805.1	625.0	740.3	1689.0	228.4	469.3
miR-133b	73.8	114.7	24.5	31.3	30.2	61.3	11.9	18.2
miR-135a	34.1	15.5	31.4	11.4	26.1	10.6	32.0	9.1
miR-136	655.7	151.4	850.8	381.8	566.9	129.5	629.4	155.5
miR-137	110.9	32.8	129.1	45.6	84.6	24.3	100.8	24.5
miR-138	83.1	36.1	87.1	7.8	72.2	16.5	63.8	16.3
miR-139-5p	132.1	41.3	155.5	8.6	142.3	20.7	124.2	33.0
miR-140	263.0	110.3	286.0	76.0	318.9	61.3	271.3	60.5
miR-141	1199.3	987.2	1624.5	1308.5	1851.6	863.5	1209.0	298.7

miR-142-3p	2560.0	1122.4	3794.9	2427.3	14979.1	15034.9	9449.6	11113.3
miR-142-5p	131.6	28.2	146.1	48.2	102.9	24.5	183.1	43.6
miR-143	12385.1	5485.6	12932.7	6274.4	13192.1	2924.0	10947.2	3303.7
miR-144	1065.4	533.2	1132.0	699.7	1292.3	426.1	806.8	382.9
miR-145	12231.4	2580.9	11225.9	6026.6	11523.7	2114.3	8883.8	2790.8
miR-146a	550.6	190.1	687.3	348.0	1042.1	653.1	800.8	331.1
miR-146b	335.5	126.9	326.6	200.7	342.7	58.7	269.4	38.6
miR-148a	4157.7	1734.1	5246.0	1587.4	4273.3	1019.1	4470.5	942.4
miR-148b	192.3	83.4	202.8	65.0	228.0	24.5	196.3	42.4
miR-149	60.2	50.7	39.9	21.4	46.7	30.0	35.0	17.9
miR-150	1319.7	222.3	1518.5	109.1	7238.5	6836.8	5225.5	6477.8
miR-151-3p	56.1	11.0	48.7	9.4	48.4	10.5	42.8	11.1
miR-151-5p	1295.6	439.2	1387.6	656.3	1314.5	269.8	1211.5	282.9
miR-152	1665.6	657.0	1869.8	669.5	1540.0	401.4	1580.7	488.7
miR-154	162.0	43.9	175.2	48.9	122.7	44.8	135.3	39.8
miR-15a	2021.2	886.4	2217.6	592.9	2281.7	267.3	2224.6	400.7
miR-15b	1074.0	353.2	1273.4	204.5	2294.2	1359.9	1814.3	1284.3
miR-16	13642.7	5659.5	14085.6	6901.9	18298.7	3473.1	14493.6	3476.7
miR-181a	1849.2	477.6	2587.2	977.7	2100.1	293.7	2008.7	497.5
miR-181b+miR-181d	37.1	16.7	28.5	19.0	34.4	4.4	20.3	12.1
miR-181c	43.2	27.8	53.8	17.7	62.1	9.8	45.9	19.8
miR-183	32.0	22.2	31.4	32.8	44.2	21.8	23.3	5.4
miR-1839-3p	43.5	27.7	20.7	12.3	50.8	20.0	29.3	10.0
miR-1839-5p	187.9	67.1	237.9	23.3	195.5	46.8	199.3	36.6
miR-185	299.5	112.5	322.7	116.5	347.0	43.9	244.9	32.8
miR-188-3p	24.5	8.1	23.8	7.7	31.5	6.9	21.7	5.2
miR-1896	214.9	40.4	188.4	33.5	189.8	22.5	170.9	19.1
miR-18a	26.0	15.0	26.2	15.1	41.8	12.0	32.2	13.5
miR-190	90.2	47.9	98.2	39.4	87.8	12.1	91.6	36.8
miR-1900	224.6	54.2	228.3	48.3	234.9	26.7	190.9	14.6
miR-1903	42.4	22.8	33.4	16.9	38.9	13.8	33.7	9.0
miR-1906	52.9	13.5	48.6	10.3	50.9	9.2	40.9	5.8
miR-190b	31.6	5.8	35.2	13.3	35.0	4.4	34.6	8.4
miR-191	429.1	120.9	449.5	216.0	518.8	81.4	443.7	116.7
miR-192	21.8	14.9	30.3	15.4	33.5	6.7	26.7	11.8
miR-1927	59.1	11.8	58.9	10.6	51.9	10.2	52.5	8.0
miR-1929	64.4	27.0	50.9	12.4	63.4	19.4	55.0	14.0
miR-193	1261.8	721.1	1202.4	324.5	1633.9	485.7	1237.4	588.4
miR-1931	124.7	132.6	102.3	59.4	96.3	116.7	28.3	30.5
miR-1937a+miR-1937b	4760.2	5133.0	2597.6	1920.2	1720.0	476.4	2690.2	2939.6
miR-1937c	5768.3	4355.5	3177.2	1960.7	2668.8	849.4	3656.2	3778.8
miR-193b	73.9	65.5	73.6	43.3	97.9	46.7	62.3	46.7
miR-194	103.3	54.4	120.7	64.1	160.9	9.2	104.0	23.8
miR-1944	9200.4	6322.1	9891.1	8147.6	8926.5	1786.2	6385.1	943.9
miR-195	873.4	411.4	768.3	400.7	1040.8	226.6	715.6	144.3
miR-1953	70.1	42.5	54.1	22.3	59.7	17.6	53.1	23.4
miR-1955	133.8	33.6	119.8	24.4	132.3	12.4	121.5	8.2
miR-1960	93.3	24.0	105.7	15.0	107.3	13.0	88.2	12.8
miR-1961	120.2	21.7	111.9	28.1	109.7	12.9	104.1	20.2

miR-1965	102.5	14.5	89.1	20.7	97.4	10.6	80.3	7.9
miR-1966	122.7	17.6	120.3	30.8	120.2	8.4	97.2	5.7
miR-1968	30.7	12.6	32.8	7.1	43.3	5.3	31.7	9.6
miR-196a	687.3	224.2	929.2	121.7	647.7	190.1	671.4	235.7
miR-196b	251.7	125.4	322.4	134.5	243.9	80.9	213.2	88.8
miR-199a-3p	17147.3	8688.4	19118.4	4938.8	15010.0	6458.6	13591.1	1409.9
miR-199a-5p	7188.9	2613.3	6774.3	3485.3	7357.6	2249.4	5840.1	1205.3
miR-19a	1120.5	553.1	1242.1	486.6	1746.0	456.5	1286.7	405.5
miR-19b	756.3	350.8	746.7	373.0	1173.6	240.2	865.1	185.0
miR-200a	565.1	435.5	580.7	429.3	823.4	360.2	624.2	242.2
miR-200b	795.2	373.2	750.2	509.8	942.3	373.9	894.1	252.8
miR-200c	446.1	210.0	469.9	358.4	552.3	222.0	521.8	161.1
miR-202-5p	23.9	18.2	17.3	4.9	15.4	8.0	22.2	9.1
miR-203	184.5	64.5	271.2	136.9	233.3	53.9	161.8	79.6
miR-204	252.2	102.1	271.7	158.2	167.1	58.4	175.8	41.5
miR-205	2308.3	1599.0	2044.2	2310.0	2471.2	1370.6	1785.1	712.8
miR-206	133.6	184.2	110.7	116.4	94.5	126.3	34.0	53.4
miR-20a+miR-20b	1982.3	883.5	2484.9	895.0	2680.2	511.4	2362.4	550.4
miR-21	12954.1	6370.2	14720.5	4474.4	14336.5	2075.6	12624.4	1430.7
miR-210	266.9	86.4	337.7	59.6	280.6	92.1	214.5	17.5
miR-2132	359.7	99.8	844.0	1225.6	868.7	590.5	437.3	241.5
miR-2134	13.9	13.1	29.2	59.3	14.3	12.9	19.3	18.0
miR-2135	28.3	17.7	27.0	17.9	25.7	23.9	42.2	18.3
miR-2137	19.4	12.9	46.9	60.1	32.0	11.7	21.1	15.3
miR-214	84.9	33.6	82.3	49.5	92.1	57.6	54.3	25.6
miR-2140	17.4	11.3	42.4	52.1	24.1	20.1	28.5	22.0
miR-2141	56.9	28.9	393.6	737.4	60.6	36.0	110.3	104.7
miR-2146	12.1	11.3	47.5	99.9	20.9	20.5	29.9	29.2
miR-218	246.9	107.5	258.7	78.3	238.4	75.2	208.7	61.2
miR-2183	147.2	70.9	153.7	57.5	176.7	59.5	134.6	43.1
miR-22	13937.4	4932.3	18069.7	4041.1	12764.9	3127.3	13138.8	3858.5
miR-221	85.0	48.1	60.4	34.4	99.6	30.3	66.3	27.1
miR-222	42.9	16.5	41.2	26.4	59.1	20.3	33.9	15.9
miR-223	1894.0	547.2	2174.4	443.2	1491.5	259.8	1580.6	165.1
miR-23a	9214.4	2993.2	10947.9	1544.2	8992.9	1734.0	8323.3	2706.0
miR-23b	1573.1	652.4	1623.1	709.7	1725.5	243.0	1277.1	225.7
miR-24	920.2	384.2	761.1	352.3	1157.2	171.2	716.8	220.7
miR-25	1441.3	421.8	2128.2	993.7	1697.7	218.6	1517.0	338.2
miR-26a	207.6	87.8	199.0	137.7	381.3	119.8	199.0	60.8
miR-26b	1183.4	595.0	1288.9	593.4	1533.1	171.5	1210.2	189.3
miR-27a	3065.8	1382.5	3929.3	367.0	3196.4	688.1	3191.1	712.1
miR-27b	65.6	24.8	154.1	178.5	73.0	18.0	51.7	5.1
miR-28	319.5	103.4	323.6	157.4	340.1	29.4	270.2	20.8
miR-296-5p	37.5	15.7	24.1	16.5	60.9	14.9	29.5	13.3
miR-297a+miR-466f+miR-669b	77.7	42.6	48.6	13.1	84.4	26.0	49.5	22.3
miR-297c	207.0	84.1	110.1	27.2	228.8	76.2	154.9	64.0
miR-29a	7102.7	2941.5	8908.7	1479.9	7936.1	452.7	7352.8	1420.2
miR-29b	4599.7	2329.6	4802.7	1469.7	5508.4	518.1	4622.9	785.7

miR-29c	4761.8	2282.5	5431.1	1327.1	5069.6	1012.7	4449.8	1137.4
miR-300	32.5	11.8	48.9	12.2	29.5	7.5	33.3	12.4
miR-301a	301.6	110.9	320.0	132.5	342.7	53.0	314.1	61.3
miR-301b	239.3	79.9	335.0	115.6	257.6	47.6	217.2	68.9
miR-30a	3617.8	1284.8	5053.0	1896.7	3937.1	776.6	3438.8	945.4
miR-30b	504.6	162.2	437.4	230.9	769.8	116.5	429.0	62.3
miR-30c	3390.2	1083.2	4042.7	398.8	3746.6	587.0	3627.0	867.8
miR-30d	1693.2	579.3	1772.2	704.7	1903.2	197.6	1523.7	263.0
miR-30e	298.0	144.9	489.9	424.3	417.6	71.3	307.2	29.1
miR-31	190.0	114.0	205.4	105.8	285.4	47.9	197.0	26.5
miR-32	444.7	255.0	473.9	241.0	612.9	67.1	419.9	88.5
miR-322	1192.3	523.6	1216.8	603.3	1188.5	210.5	1222.5	416.6
miR-324-5p	88.5	24.8	64.6	34.8	110.2	14.2	67.5	10.1
miR-326	22.4	9.7	31.7	23.5	24.9	10.5	29.1	7.7
miR-328	337.3	149.1	289.2	92.2	311.0	67.9	228.4	63.7
miR-329	195.9	51.3	203.3	70.9	137.6	51.3	161.6	56.4
miR-33	24.0	13.2	22.7	9.1	40.3	12.4	26.4	20.9
miR-331-3p	130.4	29.6	124.3	61.4	123.5	15.8	109.1	34.1
miR-335-3p	80.4	57.7	51.1	16.9	49.7	13.6	46.5	19.7
miR-335-5p	808.6	670.4	826.7	521.3	454.4	221.4	650.9	452.8
miR-338-3p	712.4	363.3	846.9	365.3	423.5	220.9	542.4	145.7
miR-338-5p	39.4	7.9	41.0	18.2	36.4	11.8	31.8	11.2
miR-340-3p	36.8	22.7	32.3	16.5	46.4	12.2	43.8	10.7
miR-340-5p	308.4	129.6	351.7	77.7	383.3	78.3	292.2	57.0
miR-342-3p	564.5	161.3	547.1	257.0	1429.7	1086.1	976.4	802.5
miR-345-5p	81.9	24.5	76.1	21.3	79.7	11.8	63.9	15.2
miR-3471	69.4	115.2	16.3	12.0	26.7	4.1	28.5	18.1
miR-34a	156.0	59.7	131.5	54.7	227.2	46.7	138.6	24.2
miR-34b-5p	32.8	23.2	24.5	17.4	30.3	9.6	21.8	13.5
miR-34c	6002.0	993.8	5389.0	2945.9	4528.8	1672.3	3456.2	2287.1
miR-350	637.7	272.8	721.3	349.3	771.9	89.3	656.2	171.2
miR-361	137.8	48.5	131.7	48.6	205.1	55.2	115.4	30.3
miR-362-3p	201.2	93.6	198.1	85.0	209.5	31.7	204.3	51.2
miR-365	4202.3	1099.7	3961.0	2014.8	4421.1	935.1	3442.9	1173.1
miR-367	24.9	10.7	35.0	18.9	34.4	5.1	24.5	8.2
miR-369-3p	115.5	56.6	123.9	33.9	95.8	37.5	100.8	31.3
miR-374	160.9	68.4	166.3	55.5	223.2	50.7	172.8	24.0
miR-376a	543.9	134.0	542.5	128.7	646.4	154.4	502.3	191.7
miR-376b	114.6	44.0	113.2	38.0	99.8	25.4	120.8	36.9
miR-376c	111.8	52.1	126.2	18.9	93.6	40.3	92.8	39.6
miR-377	145.5	25.3	148.8	14.5	136.2	21.0	129.0	31.1
miR-378	2303.7	936.3	3093.9	1403.6	2114.0	947.6	1843.8	349.2
miR-379	58.7	13.2	53.3	9.4	40.9	22.6	43.5	18.8
miR-381	40.5	17.1	56.5	16.3	31.7	8.3	47.5	7.5
miR-382	83.7	24.5	113.5	57.2	50.7	22.0	79.6	27.7
miR-410	70.1	16.0	77.8	29.8	48.3	22.7	52.8	27.8
miR-411	25.2	10.3	23.1	12.9	12.9	5.0	23.6	13.3
miR-421	21.6	5.0	16.1	9.1	21.3	6.7	19.3	7.1
miR-423-3p	315.1	78.7	280.8	65.4	413.1	72.0	273.7	57.0

miR-423-5p	186.8	57.4	148.7	53.7	208.2	35.7	139.9	33.3
miR-425	423.8	70.9	462.8	72.1	564.2	125.4	476.3	155.2
miR-429	1041.2	700.9	1314.5	1033.0	1419.9	694.3	960.1	193.8
miR-433	33.2	14.1	33.6	16.9	26.6	6.9	25.7	10.4
miR-434-3p	150.1	47.8	148.2	79.7	125.2	54.3	111.7	34.8
miR-450a-5p	268.1	124.7	307.5	106.8	331.4	27.1	239.9	75.8
miR-451	2799.3	1094.9	3356.1	1317.4	3211.7	910.3	2580.8	1317.0
miR-455	20.9	11.9	20.7	10.7	29.2	6.8	18.4	9.3
miR-466g	427.1	172.9	344.2	93.8	365.0	87.7	348.2	111.7
miR-467b	6.1	5.2	10.4	13.4	29.0	28.2	29.8	32.1
miR-467f	40.1	13.7	48.8	11.2	100.1	75.1	75.7	82.6
miR-484	180.0	53.1	148.2	80.6	249.0	39.7	144.4	49.8
miR-486	424.4	294.6	371.7	189.8	327.6	227.4	179.4	75.9
miR-489	54.8	31.8	49.6	12.5	61.6	24.8	45.2	17.7
miR-495	36.5	15.1	31.8	13.4	26.7	13.3	29.7	19.9
miR-497	316.0	150.6	269.9	111.4	378.3	93.0	241.5	66.5
miR-500	69.6	25.2	36.9	17.0	86.8	7.9	40.7	21.0
miR-501-3p	42.6	12.9	38.2	10.8	26.4	9.2	29.7	9.1
miR-503	20.2	14.6	17.4	18.2	22.0	12.2	19.2	12.0
miR-532-3p	31.4	16.2	27.5	17.0	40.1	6.1	24.2	13.9
miR-532-5p	176.0	33.2	203.2	49.3	162.0	14.8	144.4	31.6
miR-539	104.5	25.7	110.3	20.7	111.3	18.0	92.7	30.2
miR-542-5p	41.5	18.0	36.3	17.5	43.2	14.8	36.2	9.7
miR-574-3p	220.6	76.9	209.4	108.5	208.4	30.4	157.5	72.1
miR-652	55.6	10.0	62.9	21.9	54.8	10.2	51.4	12.2
miR-664	140.1	95.0	123.9	30.3	143.8	32.7	120.6	39.2
miR-669a	34.4	14.3	36.7	20.0	53.6	25.6	44.9	32.3
miR-669f	21.0	13.7	17.6	20.7	37.4	35.4	28.4	17.8
miR-672	28.3	18.4	31.4	21.3	27.3	7.6	33.2	22.4
miR-674	45.1	13.4	60.8	47.5	39.0	7.6	39.2	10.3
miR-676	49.6	5.5	55.1	21.0	46.7	14.4	48.4	24.4
miR-690	15.8	11.5	29.1	33.2	24.1	22.3	22.2	21.0
miR-691	60.3	137.2	35.7	71.8	8.0	8.5	29.3	64.9
miR-706	7.7	9.6	40.3	62.5	17.7	17.8	14.4	8.4
miR-708	236.8	51.0	221.1	68.6	278.2	49.3	224.1	93.7
miR-709	22.8	7.7	31.3	30.9	16.5	6.2	13.8	9.5
miR-720	97264.3	180772.3	88625.2	178133.3	15896.5	4728.7	68221.5	128128.8
miR-762	100.1	60.3	73.1	71.5	122.0	49.8	73.1	57.5
miR-7a	30.6	11.0	59.3	40.8	74.1	34.1	54.8	36.1
miR-804	121.8	74.2	139.0	93.5	125.6	46.9	109.8	39.8
miR-872	65.9	31.7	99.8	24.5	70.5	13.9	87.7	12.7
miR-92a	17.6	12.1	12.8	10.0	85.3	70.4	39.9	29.3
miR-93	302.7	93.0	300.0	152.2	357.5	44.1	292.6	41.7
miR-96	147.2	102.4	149.0	131.2	184.7	86.1	137.7	38.8
miR-98	301.9	115.2	353.4	129.2	355.7	63.4	258.2	46.8
miR-99a	2657.1	745.5	2895.2	914.2	2186.2	611.5	2593.3	764.8
miR-99b	794.0	250.7	745.3	392.8	848.0	168.8	625.8	202.9

* Average and SD of all miRNAs detected in MG across different groups (n=5-6/group). Data were calculated using background-subtracted and normalized miRNAs counts.

Table S6: Validated targets of the 10 deregulated miRNAs in the MG

**miRNA**	**Validated gene target**
miR-142a-5p	***Abcg1***
miR-142a-5p	***Becn1***
miR-142a-5p	***Cd28***
miR-142a-5p	***Cwc25***
miR-142a-5p	***Memo1***
miR-142a-5p	***Nmrk2***
miR-142a-5p	***Nr1d2***
miR-142a-5p	***Phf3***
miR-142a-5p	***Six2***
miR-142a-5p	***Sort1***
miR-142a-5p	***Srgap1***
miR-142a-5p	***Wnt11***
miR-142a-5p	***Wwp1***
miR-1966	***Acad9***
miR-1966	***Ppp1r16b***
miR-1	***Acta1***
miR-1	***Adar***
miR-1	***Anxa5***
miR-1	***Bdnf***
miR-1	***Calm1***
miR-1	***Calm2***
miR-1	***Cdc42***
miR-1	***Cdk9***
miR-1	***Egfr***
miR-1	***Ets1***
miR-1	***Fzd7***
miR-1	***Gata4***
miR-1	***Gja1***
miR-1	***GTF2B***
miR-1	***Hdac4***
miR-1	***Hes1***
miR-1	***Hspa1b***
miR-1	***Hspd1***
miR-1	***Igf1***
miR-1	***Igf1r***
miR-1	***Klf4***
miR-1	***Map4k3***
miR-1	***Mef2a***
miR-1	***Myh6***
miR-1	***Myocd***

miR-1	***Nfat5***
miR-1	***Nppa***
miR-1	***Pax3***
miR-1	***Pax7***
miR-1	***Pola1***
miR-1	***Rarb***
miR-1	***Rasa1***
miR-1	***Rheb***
miR-1	***Rps6***
miR-1	***Sh3bgrl***
miR-1	***Smarcb1***
miR-1	***Smarcd2***
miR-1	***Srf***
miR-1	***Tlx2***
miR-1	***Ucp2***
miR-210	***Acvr1b***
miR-210	***Agtrap***
miR-210	***Arg1***
miR-210	***Bcl2***
miR-210	***Ctla4***
miR-210	***Cxcl12***
miR-210	***Foxk1***
miR-210	***Foxp3***
miR-210	***Hif1a***
miR-210	***Il16***
miR-210	***Il2ra***
miR-210	***Inpp5d***
miR-210	***Lcp2***
miR-210	***Ncam1***
miR-210	***Ndrg1***
miR-210	***Runx3***
miR-210	***Shh***
miR-210	***Tcf7l2***
miR-210	***Tns4***
miR-210	***Trim65***
miR-210	***Ubtf***
miR-210	***Ucp2***
miR-297c	***Akap2***
miR-297c	***Asb7***
miR-297c	***Brat1***
miR-297c	***Camk1d***
miR-297c	***Casp8***

miR-297c	***Cd28***
miR-297c	***Cox15***
miR-297c	***Cpne3***
miR-297c	***Ddx6***
miR-297c	***Dnaaf5***
miR-297c	***Eif2s1***
miR-297c	***Ell2***
miR-297c	***Eno2***
miR-297c	***Etv3***
miR-297c	***Gabrb2***
miR-297c	***Gnaq***
miR-297c	***Gnb4***
miR-297c	***Has2***
miR-297c	***Htr1f***
miR-297c	***Il21***
miR-297c	***Kcnip3***
miR-297c	***Lin7a***
miR-297c	***Lrrc40***
miR-297c	***Mylk4***
miR-297c	***Ncam1***
miR-297c	***Neu1***
miR-297c	***Nrf1***
miR-297c	***Nsd2***
miR-297c	***Nsd3***
miR-297c	***Pstpip2***
miR-297c	***Ptprk***
miR-297c	***Rab3c***
miR-297c	***Rorb***
miR-297c	***Rpusd2***
miR-297c	***Sema6a***
miR-297c	***Sike1***
miR-297c	***Six4***
miR-297c	***Ski***
miR-297c	***Slc12a2***
miR-297c	***Slc1a2***
miR-297c	***Snap25***
miR-297c	***Tmco1***
miR-297c	***Tsc22d3***
miR-297c	***Ubxn2b***
miR-297c	***Uhrf1bp1l***
miR-297c	***Vdr***
miR-297c	***Vps37a***

miR-297c	***Xiap***
miR-297c	***Zc3h12a***
miR-297c	***Zeb2***
miR-30b	***Amer1***
miR-30b	***Bach2***
miR-30b	***Camk4***
miR-30b	***Cdk13***
miR-30b	***Csf1***
miR-30b	***Eno2***
miR-30b	***Gskip***
miR-30b	***Hoxb3***
miR-30b	***Ing1***
miR-30b	***Ints12***
miR-30b	***Lpar3***
miR-30b	***Mgea5***
miR-30b	***Mtdh***
miR-30b	***Neurod1***
miR-30b	***Nfatc3***
miR-30b	***Pgr***
miR-30b	***Reep4***
miR-30b	***Rrad***
miR-30b	***Serpine1***
miR-30b	***Six4***
miR-30b	***Strip1***
miR-30b	***Tnrc6b***
miR-30b	***Wdr44***
miR-324-5p	***Gjc3***
miR-324-5p	***Rcan2***
miR-324-5p	***Smo***
miR-324-5p	***Tmc8***
miR-324-5p	***Vash1***
miR-382	***Aass***
miR-382	***Akap5***
miR-382	***Lbr***
miR-382	***Trim65***
miR-382	***Xpo7***
miR-423-3p	***Cox6a2***
miR-423-3p	***Ndufb7***
miR-423-3p	***Ndufs5***
miR-500	***Aak1***
miR-500	***Bcl2l11***
miR-500	***Sin3a***

miR-500	***Ube2v2***
miR-500	***Wtap***

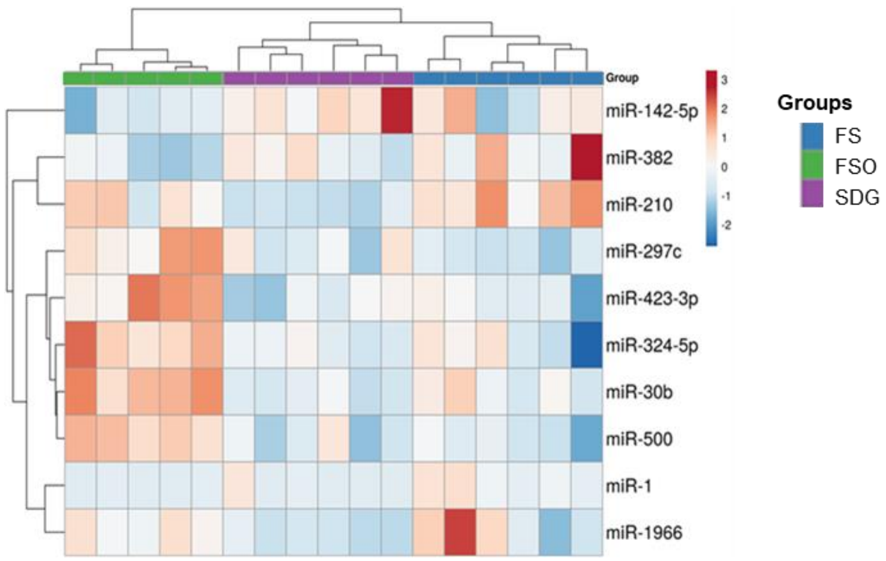

Supplementary Figure 1: Heatmap showing unsupervised hierarchical clustering of the 10 significantly deregulated miRNAs between FS, FSO and SDG groups (P-value <0.05, FDR<0.2), n=5-6/group. For each miRNA, the expression values were transformed to Z-scores, where red indicates higher expression and blue indicates lower

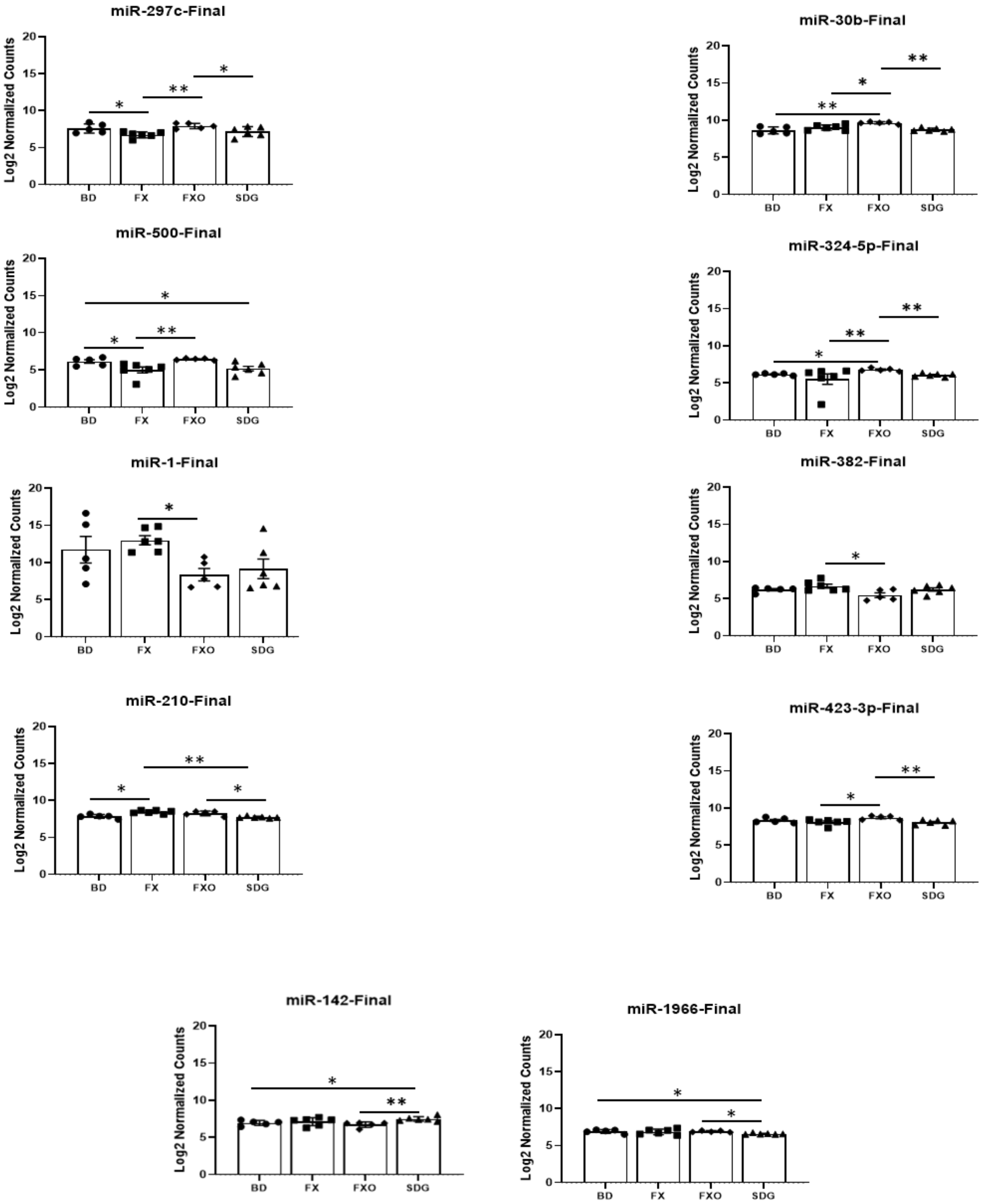

Supplementary Figure 2: Log2 normalized counts of the 10 deregulated miRNAs in response to diets. Significance assessed by ANOVA (*P<.05 and **P<.01).
